# Low canopy temperature and high stomatal conductance contribute to high grain yield of contrasting *japonica* rice under aerobic conditions

**DOI:** 10.3389/fpls.2023.1176156

**Published:** 2023-05-12

**Authors:** Wenliu Gong, Christopher Proud, Shu Fukai, Jaquie Mitchell

**Affiliations:** ^1^ School of Agriculture and Food Sciences, The University of Queensland, Brisbane, QLD, Australia

**Keywords:** aerobic rice, stomatal conductance, canopy temperature depression, photosynthetic rate, grain yield

## Abstract

Water limitation is a major concern in rice production. It has been suggested that with adapted genotypes, aerobic rice production offers the maintenance of grain yield while saving water. However, there has been limited exploration of *japonica* germplasm adapted for high-yield aerobic environments. Therefore, three aerobic field experiments with different levels of relatively high-water availability were conducted across two seasons to explore genetic variation in grain yield and physiological traits that contributed to high yield. In the first season, a *japonica* rice diversity set was explored under well-watered (WW20) conditions. While in the second season, a well-watered (WW21) experiment and an intermittent water deficit (IWD21) experiment were conducted to examine the performance of a subset of 38 genotypes selected for low (mean of −6.01°C) and high (mean of −8.22°C) canopy temperature depression (CTD). In WW20, CTD explained 19% of the variation in grain yield which was similar to the variation explained by plant height, lodging, and leaf death response to heat. In WW21, a relatively high average grain yield (9.09 t ha^−1^) was achieved, while a 31% reduction was achieved in IWD21. Compared with the low CTD group, the high CTD group had 21% and 28% higher stomatal conductance, 32% and 66% higher photosynthetic rate, and 17% and 29% higher grain yield in the WW21 and IWD21, respectively. This work demonstrated the advantage of higher stomatal conductance and cooler canopy temperature which resulted in higher photosynthetic rate and higher grain yield. Two promising genotypes with high grain yield, cooler canopy temperature, and high stomatal conductance were identified as donor genotypes for use by the rice breeding program when aerobic rice production is a target. Field screening for cooler canopies within a breeding program with high-throughput phenotyping tools would be of value for genotype selection for aerobic adaption.

## 1 Introduction

Australian temperate rice (*Oryza sativa* L.) production is traditionally based in the Riverina and grown under permanent water conditions from irrigation from river water and sometimes groundwater (Meyer, 2005). Riverina rice growers achieve the highest grain yield (GY) in the world at approximately 10 t ha^−1^ (ABARES). In the past 15 years, water productivity in Australian rice production, which is one of the most efficient rice industries in the world, has increased by 60% and the rice crop uses 50% less water than the global average (R. G. A, 2022). The high water productivity in Australian rice production was mainly achieved by the development of new cultivars with high yield and improved crop management (Humphreys et al., 2006). However, due to drought, water supply restrictions, and costs associated with irrigation water, there is a need for further improvement in water productivity. To minimize water use and maximize water productivity and GY, it has been suggested that water management strategies such as aerobic rice production should be considered.

Aerobic rice production is a system in which rice is grown in well-drained, non-flooded, and unsaturated, but well-watered conditions, and has been shown to improve water productivity (Bouman et al., 2005). In Northern China, Yang et al. (2005) found that in the aerobic experiments with 400-650 mm water input, the GY of the aerobic rice genotypes HD297 and HD502 was approximately 3 to 5.7 t ha^−1^. Fukai and Mitchell (2022a) in their review revealed that the standard aerobic production from 18 different studies had an average GY at 6.9 t ha^−1^. In Japan, the rice genotype Takanari produced similar or higher GY above 10 t ha^−1^ under aerobic conditions (10 mm of irrigation every other day) compared with flooded conditions (Kato et al., 2009). The studies mentioned above highlight that relatively high GY in aerobic production systems is a reality. As is observed in most annual cereal crops, plant height, days to heading (DTH), and harvest index are known to be large determinants of GY (Vergara et al., 1966; Kawano, 1990; Sakamoto and Matsuoka, 2008). However, aerobic rice production is a relatively new system, and the understanding of the traits contributing to the maintenance of high GY under different levels of relatively high-water availability is limited.

A recent review exploring the role of canopy temperature depression (CTD) in rice noted that genotypic variation in stomatal conductance (*g*_s_) and CTD were closely related to photosynthesis and were identified as key determinants of GY under flooded and drought conditions (Fukai and Mitchell, 2022b). Joshi et al. (2018) and Zaman et al. (2018) demonstrated that genotypes with higher *g*_s_ had higher GY under flooded and drought conditions; however, they only evaluated four and two genotypes, respectively. Examining 27 rice genotypes, lower canopy temperature was found to be associated with increased GY (*r* ^2^ = 0.63**) under drought conditions (Garrity and O'toole, 1995). Also under heat stress conditions at the flowering stage, it was found that heat-tolerant rice genotypes with lower canopy temperature achieved higher photosynthesis, higher spikelet fertility, and higher GY compared with heat-sensitive genotypes (Karwa et al., 2020). While the above studies have highlighted that high *g*_s_ and cooler canopy temperature resulted in advantages in flooded, drought, and heat stress conditions, there have been limited studies conducted under aerobic field conditions that have evaluated a large number of *japonica* germplasm. Most studies have also been conducted in regions with much lower GY potential compared with the high GY achieved in temperate Australian production. In aerobic rice production, frequent irrigation events are required, where water supply is guaranteed to maintain plant growth (Fukai and Mitchell, 2022a), and it may be hypothesized that genotypes which can maintain high CTD and *g*_s_ between irrigation events would be ideal in ensuring high GY.

Genetic variation for potential adaptation to an aerobic target environment has not been explored in rice germplasm pertinent to the Australian breeding program. Hence, the objectives of this study were to 1) evaluate the importance of CTD in determining GY in comparison to other characteristics in a predominantly *japonica* rice diversity set and 2) determine the effect of selection for high and low CTD on *g*_s_, other gas exchange parameters, and GY in aerobic conditions under different water availabilities.

## 2 Materials and methods

### 2.1 Research location

Three field experiments were conducted at the University of Queensland Research Farm at Gatton, Queensland, Australia (17°33′S, 152°17′E). One well-watered experiment (WW20) was conducted from October 2019 to April 2020 (year 1). Two experiments were conducted from November 2020 to May 2021 (year 2): well-watered (WW21) and intermittent water deficit (IWD21).

### 2.2 Genetic material and experimental designs

A *japonica* rice diversity set consisting of 241 genotypes including a mix of lowland and upland genotypes compiled by the New South Wales Department of Primary Industries was used in the WW20 experiment. The genotypes were from 35 countries, with 63 genotypes from Australia and 10 genotypes of unknown origin. The WW20 experiment was arranged in a partially replicated design with 44 genotypes replicated once while the remaining had two replications. The R package *DiGGer* was used to generate a partially replicated row-column design (Coombes, 2009).

Year 2 experiments utilized 35 genotypes consisting of the highest and lowest CTD genotypes based on WW20 following the selection strategy described below (**Supplementary Table 1**). The selection was conducted to remove the confounding effect of plant height and DTH. There were also three check genotypes included. A row-column design with three replications was utilized for WW21 and IWD21. The 35-genotype selection was based on the genotypes which were shorter than 120 cm, yielded higher than 4.5 t ha^−1^, and reached the heading stage between 80 and 110 days after sowing (DAS). Of the remaining 113 genotypes, the high CTD group consisted of 19 genotypes with CTD <-7.3°C, and the low CTD group consisted of 16 genotypes with CTD >-6.5°C. Apo, Takanari, and 55A were included in year 2 as checks. Apo was an aerobic standard genotype (Vijayaraghavareddy et al., 2020). Takanari is a well-known standard for high *g*_s_ and high yield (Adachi et al., 2019), and 55A is a breeding genotype derived from Reiziq/Tachiminori and had been observed to have high CTD (pers. comm. C. Proud).

Furthermore, in year 2, among the 38 genotypes, a subset of 15 genotypes was selected to measure the photosynthetic rate and transpiration rate. To further narrow the selection window, for genotypes that reached the heading stage between 80 and 105 days after sowing, the high CTD group had seven genotypes with CTD <-7.7°C and the low CTD group had five genotypes with CTD >-6.5°C. In total, there were five and seven genotypes from the low and high CTD groups, respectively. Apo, Takanari, and 55A were included as checks in the subset.

### 2.3 Crop management and irrigation

In all experiments, seeds were drill sown using a tractor-mounted cone seeder and consisted of 7 rows by 2 m with an inter-row spacing of 0.22 m. A seeding rate of 130 kg ha^−1^ at a depth of 3–4 cm was used in year 1. Due to the high plant density in WW20 which exceeded our target establishment and in which lodging occurred, a lower seed rate at 60 kg ha^−1^ at a depth of 5 cm was used in year 2. WW20 was sown on 17 October 2019, and WW21 and IWD21 were sown on 6 November 2020. In WW20 and WW21, irrigation was applied thrice weekly with 32 and 20 mm, respectively, *via* an overhead boom. In IWD21, the irrigation was applied mostly twice weekly with 20 mm *via* an overhead boom.

### 2.4 Measurements

#### 2.4.1 Plant density

A 33-cm ruler was randomly placed between rows in two positions per plot, and the number of seedlings on either side of the ruler (i.e., 4 rows in total) was counted to determine the number of seedlings m^−2^. Plant density was measured between 18 and 23 DAS for the three experiments.

#### 2.4.2 Canopy temperature depression

The canopy and air temperature were measured from 60 to 78 DAS in WW20 (six times) and from 60 to 70 DAS in WW21 (two times) and IWD21 (three times) coinciding with the pre-heading period (before 80% of genotypes reached 50% heading). Canopy temperature was measured by a handheld infrared thermometer AGRI­THERM II™ (100L, Everest Interscience, USA) between 12:00 and 14:00 by pointing at the canopy at an angle of 20°–45° of each plot on consistently sunny days. Air temperature and relative humidity were recorded by a handheld thermohygrometer (RS PRO, Australia) every five plots. Canopy temperature depression (°C) was the difference between canopy temperature to air temperature: CTD = *T*_canopy_ − *T*_air_.

#### 2.4.3 Stomatal conductance (*g*_s4_)

An AP4 Porometer (Delta-T Devices, Cambridge) was used to measure stomatal conductance (*g*_s4_, mmol m^−2^ s^−1^) from 60 to 70 DAS in WW21 (three times) and IWD21 (five times) coinciding with the pre-heading period. The porometer measured the *g*_s4_ of the abaxial side of the youngest fully expanded leaf by clipping the sensor head onto a leaf on clear sunny days between 10:00 and 12:00. On each measurement occasion, the mean of two plants was used to determine *g*_s4_ for each plot.

#### 2.4.4 Other gas exchange parameters

Measurements of leaf photosynthetic rate (*A*, µmol m^−2^ s^−1^), transpiration rate (TR, mmol m^−2^ s^−1^), and stomatal conductance (*g*_s68_, mmol m^−2^ s^−1^) were recorded on 15 genotypes using a portable photosynthesis meter LI-6800 (LI-COR, USA) which was set at a CO_2_ concentration of 410 µmol mol^−1^, light intensity at 2,000 µmol mol^−2^ s^−1^, air temperature at 30°C, relative humidity at 85%, and flow at 500 µmol s^−1^. Intrinsic water use efficiency (WUE_i_, μmol mol^−1^) was calculated by dividing *A* by *g*_s68_ (Condon et al., 2002). The LI-6800 measured the gas exchange parameters of both the adaxial and abaxial sides of the leaf. Measurements were conducted at 92 DAS in WW21 and at 72 and 73 DAS in IWD21.

#### 2.4.5 Leaf death score

Leaf death was first observed at 54 DAS after a heat wave in WW20. There was no obvious leaf death in WW21 and IWD21. Leaf death score (LDS) was visually recorded based on the Standard Evaluation System for drought score (IRRI, 2010). The score ranged from 0 to 9, with 0 indicating no leaf death and 9 indicating that all leaves were dead. LDS was recorded between 12:00 and 14:00 on three occasions between 64 and 82 DAS in WW20, and the mean across three measurements was used.

#### 2.4.6 Phenology, plant height, grain yield, and yield components

The days required for 50% of the heads to reach 50% extrusion were determined on a plot basis. Plant height (PH, cm) was measured at maturity. Plant lodging score was recorded at maturity only in WW20, as there was barely any lodging in year 2. The lodging score ranged from 1 to 5, where a score of 1 indicated that plants were not lodged and a score of 5 indicated that plants were fully lodged. Grain yield (t ha^−1^) was determined as each plot reached physiological maturity. The panicles of the middle 4 rows × 1 m of each plot were harvested manually for GY determination (expressed at 14% moisture). All panicles were put through a Wintersteiger thresher (LD350) to separate the grains which were oven-dried at 35°C. A representative grab subsample of approximately 30 tillers was taken at the ground level and subsequently partitioned into panicles and stems. Panicle harvest index (PHI) was determined by dividing panicle weights with the sum of the panicle and stem weights in WW20. Panicles were threshed and the harvest index (HI) was determined as the proportion of grain weight to total biomass (30 tillers) in WW21 and IWD21. Stem and panicle numbers were quantified. Spikelets per panicle and the thousand grain weight were determined by counting grain number with a Contador 2 seed counter (PFEUFFER GmbH, Germany). Grain number m^−2^ was calculated by dividing GY by the thousand grain weight.

### 2.5 Statistical analysis

For year 1, a multiplicative linear mixed model was used for the analysis and was implemented in *ASReml-R* in the R environment (V4.0.3) (Butler et al., 2009). The best spatial model was fitted for each trait (Gilmour et al., 1997). For year 2, the *SpATS* package was used for the analysis and was implemented in the R environment (V4.0.3) (Rodríguez-Álvarez et al., 2017). In both years, genotype was treated as both a random effect to estimate heritability and a fixed effect to obtain the best linear unbiased estimates (BLUEs). According to Gong et al. (in preparation), genotypes performed relatively consistent in physiological traits across different measurement days. Therefore, in this study, a repeated measures analysis was undertaken to obtain BLUEs across measurement events using the *lme4* package (Bates et al., 2015) in the R environment (V4.0.3) for CTD in all experiments, *g*_s4_ in WW21 and IWD21, and other gas exchange parameters in IWD21, with measurement events treated as a random factor.

An independent sample *t*-test model was used for the *t*-test analysis for CTD, gas exchange parameters, GY, and yield component traits between the low and high CTD groups using the Statistical Tool for Agricultural Research (V 2.0.1).

Principal component analysis (PCA) by singular value decomposition using the scaled and centered BLUEs per environment was conducted. A PCA biplot was generated using the *tidyverse* (Wickham, 2017), *ggrepel* (Slowikowski, 2020), and *ggpubr* (Kassambara, 2020) packages in the R environment (V4.0.3).

## 3 Results

### 3.1 Weather

**Figure 1:**
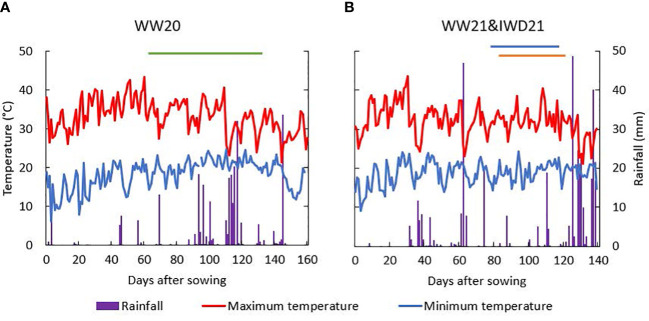
Rainfall and maximum and minimum temperatures (°C) during the experimental periods: **(A)** the well-watered experiment in the 2019–2020 (WW20) season and **(B)** the well-watered (WW21) and intermittent water deficit (IWD21) experiments in the 2020–2021 season [the heading period was indicated by a line: green (WW20), blue (WW21), and orange (IWD21)].

In year 1, the maximum temperature was high from 30 to 60 DAS, with 16 days in total that had a maximum temperature higher than 38°C. The heading stage was between 62 and 136 DAS in WW20, and during this period, the maximum temperature varied from 23.1°C to 40.7°C. Of a total rainfall of 263 mm during the growing period, 194 mm fell between 90 and 120 DAS (**Figure 1A**). In year 2, the highest maximum temperature was 43.5°C at 30 DAS. The total rainfall was 383 mm (**Figure 1B**) with an average daily solar radiation during a growing period of 21.6 MJ m^−2^ (data not shown).

### 3.2 Total water input and evapotranspiration

**Figure 2:**
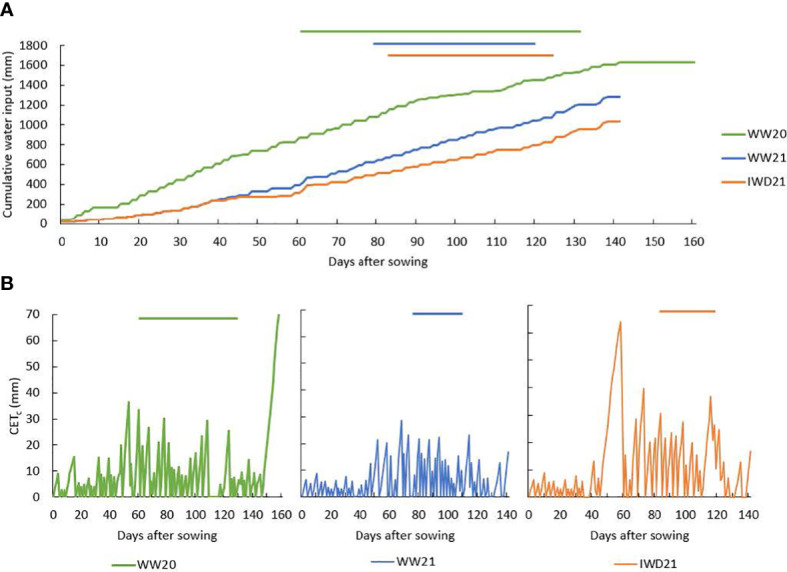
**(A)** Cumulative water input (irrigation and rainfall, mm) and **(B)** cumulative inter-rainfall/irrigation crop evapotranspiration (CET_c_) at different days in the well-watered (WW20—green, WW21—blue) and intermittent water deficit (IWD21—orange) experiments; the straight lines above the graph indicate the heading period of each experiment.

Total irrigation was 1,410 mm in WW20, 896 mm in WW21, and 648 mm in IWD21. Total water input (irrigation + rainfall), calculated until 10 days before the last harvest, was 1,647 mm in WW20, 1,280 mm in WW21, and 1,032 mm in IWD21 (**Figure 2A**). As a measure of soil water deficit during crop growth, the daily crop evapotranspiration (ET_c_) was calculated using reference evapotranspiration (ET_o_) from SILO (2022), which is a database of Australian climate data, and the crop coefficients (*k*), which were determined by Lal et al. (2012) in irrigated rice (ET_c_ = ET_o_ × *k*). The crop coefficient was 0.4, 0.86, and 1.17 from 0 to 30 DAS, from 31 to 60 DAS, and from 61 to 100 DAS, respectively. It was assumed that soil water was lost at potential ET_c_ every day and rain/irrigation reduced soil water deficit to zero. The cumulative inter-rainfall/irrigation evapotranspiration (CET_c_) was set to zero on the day of rainfall/irrigation, and ET_c_ was added from the following day to determine CET_c_. The CET_c_ was mostly less than 10 mm in the first 50 days in all three experiments (**Figure 2B**). In WW21, the maximum CET_c_ was mostly under 10 mm. In WW20, there were four occasions where CET_c_ was greater than 28 mm during the heading period. In IWD21, there was an increasing CET_c_ from 40 to 60 DAS, with relatively high CET_c_ exceeding 60 mm approximately 60 DAS which coincided with the pre-heading period. Thereafter and coinciding with the heading period, the maximum CET_c_ of 30 mm was maintained except at 120 DAS when it reached 40 mm.

### 3.3 Traits of importance in year 1

**Figure 3:**
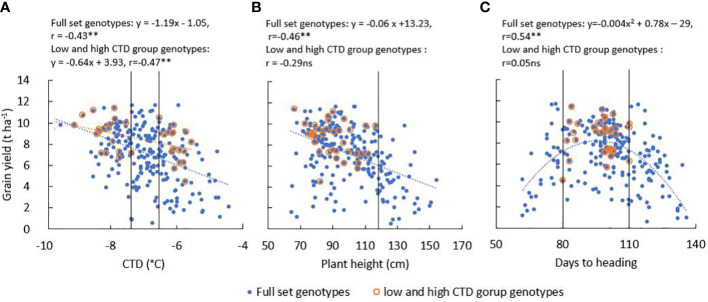
The relationship between grain yield and **(A)** canopy temperature depression (CTD), **(B)** plant height, and **(C)** days to heading of the full diversity set genotypes (*n* = 241) and high and low CTD group genotypes (*n* = 35); the black vertical lines in each graph showed the selection criteria thresholds for the high and low CTD groups. (**, P<0.01; ns, not significant).

There was significant (*P* < 0.01) genotypic variation for all measured traits in WW20 (**Table 1A**). The mean GY was 7.16 t ha^−1^ in WW20, and 51% of the genotypes had GY between 7 and 11 t ha^−1^. Most genotypes had CTD from −6°C to −8°C. Leaf death was observed in WW20; however, more than half of the genotypes had low LDS between 1 and 3. LDS was positively associated with CTD (*r* = 0.47**). GY was mostly associated with grain number m^−2^ and PHI in the traits measured in WW20 (**Table 1B**). Correlation coefficients were similar varying from −0.38** to −0.47** between GY and various traits including CTD (**Figure 3A**), lodging score, LDS, and plant height (**Figure 3B**). While the correlation coefficient between GY and DTH was not significant (*r* = 0.10 ns), there was a quadratic relationship between them in the full diversity set in WW20 (**Figure 3C**). To remove the effect of plant height and DTH and to further evaluate the importance of CTD in relation to GY, a subset of contrasting CTD genotypes was selected (criteria described in the methods). The mean GY for the subset was 8.57 t ha^−1^. The subset consisted of 19 genotypes with the highest CTD of −8.22°C on average and 16 genotypes with the lowest CTD of −6.01°C on average. Based on this WW20 subset, there was no significant relationship between GY and plant height, nor DTH, while a significant negative relationship between GY and CTD (*r* = −0.47**) remained. In the subset, there was a highly significant group difference for GY with the high CTD group 16% higher than the low CTD group, and the high CTD group had significantly higher grain number m^−2^ and lower LDS than the low CTD group (**Table 1A**).

**Table 1:** (A) The high canopy temperature depression (CTD, °C) group mean, low CTD group mean, subset including the low and high CTD group genotype mean, the full set mean, and heritability (*H* ^2^) of grain yield (GY, t ha^−1^), grain number m^−2^ (GNM), lodging score (LS), panicle harvest index (PHI), plant height (PH, cm), days to heading (DTH), leaf death score (LDS), CTD, and plant density (PD, no. plant m^−2^); (B) the correlation coefficient between different traits in the well-watered experiment (WW20) of 241 genotypes (ns, not significant; *,*P* < 0.05; **,*P* < 0.01).

(A)	GY	GNM	LS	PHI	PH	DTH	LDS	CTD	PD
High CTD mean (*n* = 19)	9.13*	41,249**	1.50 ns	0.47 ns	90 ns	98 ns	1.53*	−8.22**	312*
Low CTD mean (*n* = 16)	7.90	34,006	1.51	0.46	91	96	2.23	−6.01	242
Subset mean (*n* = 35)	8.57**	38,058**	1.51**	0.46**	90**	97**	1.85**	−7.21**	281**
									
Full set mean (*n* = 241)	7.16**	32,856**	1.82**	0.42**	100**	100**	2.15**	−6.93**	300**
*H* ^2^	0.83	0.81	0.76	0.80	0.93	0.97	0.87	0.66	0.63
									
**(B)**	GY	GNM	LS	PHI	PH	DTH	LDS	CTD	
GNM	0.80**								
LS	−0.38**	−0.30**							
PHI	0.72**	0.64**	−0.28**						
PH	−0.46**	−0.35**	0.60**	−0.59**					
DTH	0.10 ns	−0.01 ns	0.17**	−0.42**	0.50**				
LDS	−0.47**	−0.42**	0.16*	−0.39**	0.24**	−0.11 ns			
CTD	−0.43**	−0.46**	0.03 ns	−0.26**	0.08 ns	−0.15*	0.47**		
PD	−0.05 ns	0.04 ns	0.09 ns	−0.19**	0.06 ns	0.16*	0.19**	−0.17**	

### 3.4 CTD and gas exchange parameters in year 2

Significant (*P* < 0.01) genotypic variation existed for all CTD and gas exchange parameters in year 2 (**Table 2**). There were significant group differences for CTD and gas exchange parameters in the two experiments except for WUE_i_. The mean CTD was −6.01°C and −4.84°C for WW21 and IWD21, respectively. The mean CTD in the high CTD group was 18% and 22% higher than the CTD in the low CTD group in IWD21 and WW21, respectively. Sherpa, YRF210, and Lemont_(Y2) had high mean CTD averaged across the two experiments from −5.71°C to −6.48°C. The mean *g*_s4_ in the high CTD group was 21% and 28% higher than in the low CTD group in the two experiments. The well-known high *g*_s_ genotype Takanari was among the highest mean *g*_s4_ across the two experiments, which was similar to YRF210, 55A, and Lemont_(Y2) which had the highest *g*_s4_ from 275 to 323 mmol m^−2^ s^−1^ across WW21 and IWD21. The mean *A* was 21.85 and 17.15 µmol m^−2^ s^−1^ for WW21 and IWD21, respectively. In each experiment, there was a significant difference in the mean *A* between the high and low CTD groups, with the mean *A* in the high CTD group 32% and 66% higher than the mean *A* in the low CTD group in WW21 and IWD21, respectively. A similar trend was also found in TR and *g*_s68_. The high CTD group had significantly higher mean TR (41% and 76%) and mean *g*_s68_ (46% and 86%) than the low CTD group in the two experiments. The above-identified high CTD and high *g*_s4_ genotypes YRF210 and Lemont_(Y2) had consistently high mean *A*, TR, and *g*_s68_ and low WUE_i_ across the two experiments. Hence, the selection of high CTD showed consistency in maintaining higher *g*_s_, higher *A*, and higher TR in different water availabilities.

**Table 2:** Mean of the high and low canopy temperature depression (CTD, °C) group mean, overall mean, selected individual genotypes’ value, and heritability (*H* ^2^) of genotypes for CTD, stomatal conductance measured by AP4 (*g*_s4_, mmol m^−2^ s^−1^), photosynthetic rate (*A*, µmol m^−2^ s^−1^), transpiration rate (TR, mmol m^−2^ s^−1^), stomatal conductance measured by LI-6800 (*g*_s68_, mmol m^−2^ s^−1^), and intrinsic water use efficiency (WUE_i_, μmol mol^−1^) in the well-watered (WW21) and intermittent water deficit (IWD21) year 2 experiments (ns, not significant; **,*P* < 0.01).

Group		CTD (*n* = 38)		*g*_s4_ (*n* = 38)	
		WW21	IWD21	WW21	IWD21
	High CTD mean (*n* = 19)	−6.53**	−5.14**	254**	252**
	Low CTD mean (*n* = 16)	−5.35	−4.35	183	197
	Overall mean (*n* = 38)	−6.01**	−4.84**	226**	231**
Check	55A	−6.67	−5.31	277	273
Check	Apo	−6.18	−5.34	294	252
Check	Takanari	−6.09	−4.87	272	286
High CTD	Lemont_(Y2)	−7.47	−5.48	304	281
High CTD	Sherpa	−6.15	−5.26	207	286
High CTD	YRF210	−7.21	−5.37	338	309
	*H* ^2^	0.73	0.78	0.84	0.79
					
		A (*n* = 15)		TR (*n* = 15)	
		WW21	IWD21	WW21	IWD21
	High CTD mean (*n* = 7)	24.38**	20.90**	5.72**	4.10**
	Low CTD mean (*n* = 5)	18.42	12.6	4.05	2.33
	Overall mean (*n* = 15)	21.85**	17.15**	5.04**	3.30**
Check	55A	24.47	20.44	6.5	3.75
Check	Apo	18.43	9.66	4	2.12
Check	Takanari	22.08	17.89	4.86	3.48
High CTD	Lemont_(Y2)	26.04	25.21	5.35	5.39
High CTD	YRF210	24.63	22.73	6.06	4.33
	*H* ^2^	0.86	0.86	0.78	0.86
					
		*g*_s68_(*n* = 15)		WUE_i_ (*n* = 15)	
		WW21	IWD21	WW21	IWD21
	High CTD mean (*n* = 7)	391**	261**	64.78 ns	82.33 ns
	Low CTD mean (*n* = 5)	268	140	73.87	93.07
	Overall mean (*n* = 15)	339**	206**	68.1**	86.56**
Check	55A	419	234	62.98	88.11
Check	Apo	275	118	67.4	82.37
Check	Takanari	311	210	68.36	86.32
High CTD	Lemont_(Y2)	421	345	61.28	72.52
High CTD	YRF210	415	282	60.38	85.21
	*H* ^2^	0.79	0.86	0.54	0.48

### 3.5 Grain yield and yield component traits in year 2

There was significant genotypic variation in GY and yield components in year 2 (**Table 3**; **Supplementary Table 2**). The mean GY was 9.09 t ha^−1^ in WW21, with most genotypes achieving between 7 and 11 t ha^−1^. Compared with WW21, there was a 31% reduction in mean GY in IWD21. There was a significant difference in the mean GY between the high and low CTD groups, with the GY of the high CTD group being 17% and 29% higher than the low CTD group in WW21 and IWD21, respectively. WW21 had a higher grain number m^−2^ and HI than IWD21. There were also significant group differences between the high and low CTD groups for grain number m^−2^ with 25% and 35% increases in the high compared with the low CTD group in WW21 and IWD21, respectively. There were significant group differences for HI between the high and low CTD groups in WW21 but not in IWD21. Averaged across the two experiments, the Australian standard genotype Sherpa achieved the highest GY at 10.83 t ha^−1^, while the previously reported high GY genotype Takanari achieved 9.50 t ha^−1^. Lemont_(Y2) and YRF210, identified earlier to achieve high CTD and high *g*_s_, produced high GY at 9.69 and 9.99 t ha^−1^ averaged across the two experiments.

**Table 3:** Mean of the high and low canopy temperature depression (CTD, °C) group mean, overall mean, selected individual genotypes’ value, and heritability (*H* ^2^) of genotypes for grain yield (GY, t ha^−1^), harvest index (HI), and grain number m^−2^ (GNM) in the well-watered (WW21) and intermittent water deficit (IWD21) year 2 experiments (ns, not significant; *P < 0.05; **P < 0.01).

		GY		HI		GNM	
		WW21	IWD21	WW21	IWD21	WW21	IWD21
High CTD mean (*n* = 19)		9.77*	6.94**	0.48*	0.45 ns	46,597*	35,422*
Low CTD mean (*n* = 16)		8.37	5.36	0.45	0.39	37,352	26,183
Overall mean (*n* = 38)		9.09**	6.25**	0.46**	0.41**	42,937**	31,547**
Check	55A	7.11	6.69	0.41	0.37	37,360	32,971
Check	Apo	7.59	5.16	0.32	0.42	47,479	31,710
Check	Takanari	10.96	8.05	0.42	0.55	63,791	42,183
Low	Lemont_(Y2)	11.35	8.02	0.48	0.53	55,210	37,615
Low	Sherpa	11.55	10.11	0.46	0.49	57,801	50,698
Low	YRF210	10.06	9.91	0.51	0.49	41,600	46,744
	*H* ^2^	0.85	0.69	0.70	0.77	0.81	0.67

### 3.6 Factors determining grain yield in year 2

**Figure 4:**
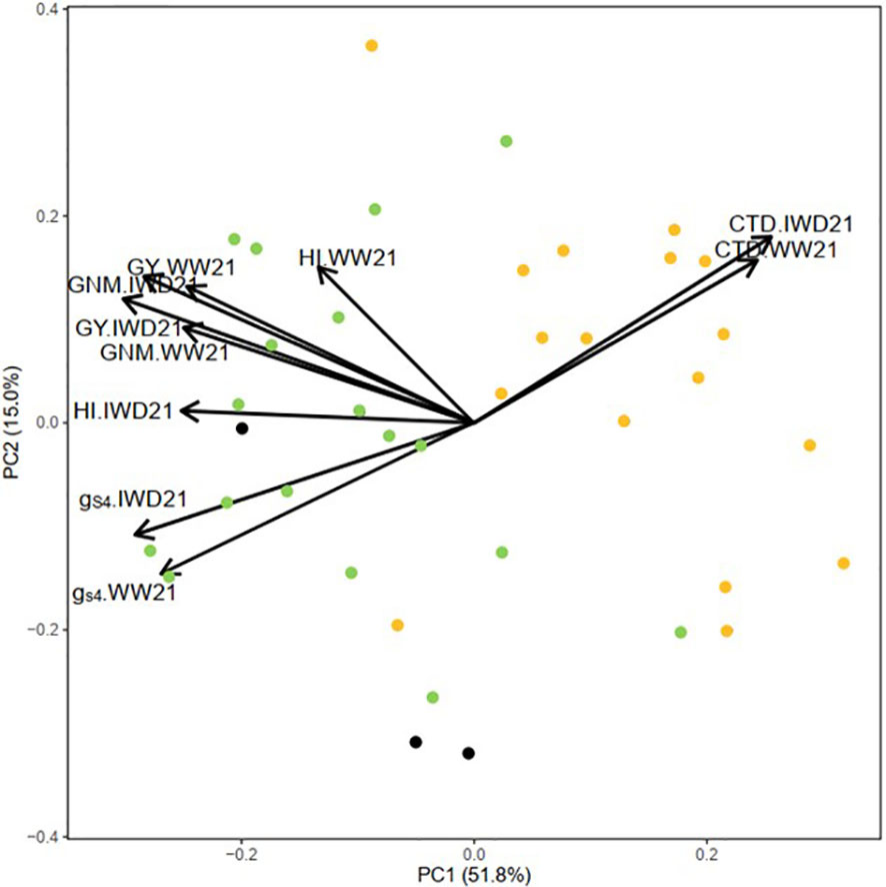
Principal component analysis (PCA) of canopy temperature depression (CTD), stomatal conductance measured by AP4 (*g*_s4_), grain yield (GY), harvest index (HI), and grain number m^−2^ (GNM) in the well-watered (WW21) and intermittent water deficit (IWD21) experiments of 38 genotypes (individual genotypes are represented in different colors: green, high CTD genotype; orange, low CTD genotype; black, check genotype).

According to **Figure 4**, the first and second principal components explained the variance of 51.8% and 15.0%, respectively. The figure showed the consistency of CTD, *g*_s4_, GY, HI, and grain number m^−2^ among the two experiments. The high CTD group clustered together with high *g*_s4_, high GY, high HI, and high grain number m^−2^.

In the two experiments in year 2, *g*_s4_ was highly significantly correlated with CTD (*r* = −0.69** and −0.70**, **Table 4**). There was a significant and moderate correlation between *g*_s4_ in relation to GY and grain number m^−2^ in the two experiments. There was also a significant correlation between CTD in relation to GY and grain number m^−2^ in the two experiments. There was an interesting trend that there was no significant correlation between HI in relation to *g*_s4_ and CTD in WW21, while the correlation was significant in IWD21. The *A*, TR, and *g*_s68_ of the 15 genotypes were negatively correlated with CTD and positively correlated with *g*_s4_. GY was significantly positively correlated with *A* in the two experiments (*r* = 0.53* and 0.56*). HI was significantly correlated with *A*, TR, and *g*_s68_ in IWD21, while the correlation was not significant in WW21. Most of the correlations between DTH, plant height, and other yield component traits in relation to CTD and *g*_s4_ in 38 genotypes or gas exchange parameters in 15 genotypes were not significant in year 2 (data not shown). In summary, by evaluating the relationship between yield components and physiological traits, higher *g*_s4_ could promote cooler canopy temperature and higher *A* and TR and thus achieve the higher GY by improving the HI and grain number m^−2^.

**Table 4:** The correlation coefficient between grain yield (GY), mean canopy temperature depression (CTD), mean stomatal conductance measured by AP4 (*g*_s4_), harvest index (HI), and grain number m^−2^ (GNM) of 38 genotypes and the correlation coefficient between the mean photosynthetic rate (*A*), transpiration rate (TR), and stomatal conductance measured by LI-6800 (*g*_s68_) in relation to CTD, *g*_s4_, GY, HI, and GNM of 15 genotypes in the well-watered (WW21) and intermittent water deficit (IWD21) experiments (ns, not significant; *P < 0.05; **P < 0.01).

WW21	GY	CTD	*g*_s4_	HI	GNM
CTD	−0.34*				
*g*_s4_	0.35*	−0.69**			
HI	0.39*	−0.21 ns	0.08 ns		
GNM	0.77**	−0.33*	0.38*	0.09 ns	
*A*	0.53*	−0.68**	0.62*	0.33 ns	0.45 ns
TR	0.45 ns	−0.56*	0.64**	0.31 ns	0.35 ns
*g*_s68_	0.50 ns	−0.58*	0.66**	0.25 ns	0.36 ns
					
IWD21	GY	CTD	*g*_s4_	HI	GNM
CTD	−0.37*				
*g*_s4_	0.51**	−0.70**			
HI	0.53**	−0.47**	0.54**		
GNM	0.94**	−0.33*	0.53**	0.59**	
*A*	0.56*	−0.74**	0.65**	0.61*	0.43 ns
TR	0.51 ns	−0.74**	0.64**	0.63*	0.44 ns
*g*_s68_	0.51 ns	−0.73**	0.64**	0.61*	0.42 ns

## 4 Discussion

### 4.1 Grain yield in aerobic conditions

In the current study, the average GY achieved in WW21 was 9.09 t ha^−1^, and a high GY was also achieved in WW20 at 8.57 t ha^−1^ in the subset. This result was congruent with the findings of Kato et al. (2009) who found a high mean GY of approximately 7.5 to 9.4 t ha^−1^ using sprinkler irrigation. The average Australian industry rice grain yield under flooded conditions for 2021–2022 was 9.8 t ha^−1^ (ABARES), and the GY achieved in the current well-watered aerobic conditions was only approximately 1 t ha^−1^ lower. Compared with the GY in WW21 in year 2, the mean of 38 genotypes decreased by 31% in IWD21. The reduction in GY was mainly due to less cumulative water input and water deficit developed during the pre-heading period in IWD21. The Australian standard genotype Sherpa had GY higher than 10 t ha^−1^ in the current aerobic experiments, which was similar to the 5-year grower average yields (10.3 t ha^−1^) under flooded conditions according to the Sherpa Growing Guide by NSW DPI (Dunn, 2020). Takanari achieved GY of 8.05 and 10.96 t ha^−1^ in the two experiments in year 2, which is similar to that established by Kato and Katsura (2014) with the GY of Takanari from 9.2 to 11.4 t ha^−1^ in aerobic conditions. The findings of the current study have highlighted that there is potential for aerobic rice production with relatively high grain yield achieved despite non-flooded conditions.

### 4.2 Importance of CTD in determining grain yield compared with other characteristics in a diversity set

In WW20, when evaluating a *japonica* rice diversity set of 241 genotypes, it was found that CTD explained 19% of the variation in GY. Additional traits that contributed to GY such as plant height and lodging score in the current aerobic conditions were also reported in other studies (Hitaka, 1969; Sakamoto and Matsuoka, 2004; Lafitte et al., 2007). The quadratic relationship between DTH and GY indicated that the optimal time for heading was between 80 and 110 days after sowing in the current study. Leaf death was only observed after a heat stress event, and genotypes with warmer canopy temperatures were more likely to have leaf death with a correlation coefficient of 0.48** between CTD and leaf death score. LDS was negatively correlated with GY in the diversity set. Lower canopy temperature has been found to enhance the ability to have lower panicle temperature and higher spikelet fertility under heat stress conditions in rice (Yan et al., 2012). Compared with other traits, CTD was found to have a similar strength correlation coefficient with GY. It should be noted that the diversity set consisted of genotypes with large variations in height and DTH. In the current aerobic environment to maximize GY, genotypes with short to medium duration and shorter plant height are required. Therefore, a subset of lines was utilized in year 2, to minimize the confounding effect of plant height and DTH on the evaluation of the effect of CTD when grown under two relatively high-water availability aerobic conditions.

### 4.3 The effect of the selection of contrasting CTD on grain yield and gas exchange parameters

Within narrower days to heading and plant height range and by selecting genotypes from the diversity set with the highest and lowest CTD in year 2, the high CTD group resulted in significantly higher GY and grain number m^−2^ compared with the low CTD in the two experiments. This finding indicated the importance of CTD in contributing to high GY in different aerobic conditions. Previously, CTD was found to be an important factor that contributed to GY in rice under more stressed conditions compared with flooded conditions. Melandri et al. (2019) found that canopy temperature was negatively correlated with GY (*r* = −0.48**) in 293 rice *indica* genotypes under drought conditions, while the correlation was weaker under flooded conditions (*r* = 0.20**). In the study of Torres and Henry (2018), the canopy temperature at flowering was significantly correlated with grain yield (*r* = 0.74**) in the dry season utilizing 11 rice genotypes, while no significant correlation was found in the wet seasons. This current study has identified the significant yield advantages conferred by higher CTD in the aerobic experiments, and this was for the first time reported in high-yielding aerobic production systems.

The yield advantages conferred by high CTD were related to the concomitant benefits on the gas exchange parameters. CTD group performance for low and high CTD was consistent across the experiments in the study. Also, for the gas exchange parameters examined in year 2, the *g*_s4_ of the high CTD group was significantly higher than that of the low CTD group in the two experiments, and furthermore, the high CTD group was associated with greater *A* and TR. The relationship between CTD and gas exchange parameters in rice was previously reported utilizing a limited number of genotypes (Takai et al., 2010; Gao et al., 2015). In the current experiments with 38 genotypes, *g*_s4_ was positively correlated with *A*, GY, and grain number m^−2^. These results suggest that higher *g*_s_ and cooler canopy temperature are advantageous for aerobic rice production, leading to higher *A* and grain yield. To our knowledge, this is the first study to report that the selection of genotypes based on contrasting CTD would identify genotypes that consistently maintain significant group differences in *g*_s_ which translated to higher *A* and GY in aerobic rice production. Furthermore, in the high CTD group, several genotypes such as YRF210 and Lemont_(Y2) achieved high GY in all experiments, and they were also able to maintain high CTD and *g*_s4_. Compared with the well-known high *g*_s_ and GY genotype Takanari, YRF210 and Lemont_(Y2) showed similar and higher performance in *g*_s_ and GY in the aerobic conditions. The two promising genotypes YRF210 and Lemont_(Y2) have entered into the breeding program as donors for aerobic adaptation.

## 5 Conclusion

In conclusion, significant genotypic variation existed for GY within a *japonica* diversity set when grown under aerobic conditions. CTD was considered an important characteristic in which the correlation coefficient with GY (*r* = −0.43**) was similar to that of plant height, lodging, and leaf death (*r* = −0.38** to −0.47**). The selection for higher CTD resulted in genotypes that maintained higher *g*_s_ and higher *A* and thus resulted in higher GY under different water availabilities. Promising donors with high GY, cooler canopy temperature, and high *g*_s_ were identified from this study and have been subsequently incorporated into the breeding program. The selection of genotypes with high CTD and high *g*_s_ could be used in rice breeding programs to achieve improvement in adaptation to aerobic production.

## Data availability statement

The raw data supporting the conclusions of this article will be made available by the authors, without undue reservation.

## Author contributions

WG: conceptualization, investigation, formal analysis, software, and writing—original draft. CP: conceptualization, methodology, software, formal analysis, and writing—review and editing. SF: conceptualization, writing—review and editing, and supervision. JM: conceptualization, writing—review and editing, supervision, and funding acquisition. All authors contributed to the article and approved the submitted version.
