# Supplementary material for: Low canopy temperature and high stomatal conductance contribute to high grain yield of contrasting japonica rice under aerobic conditions
Source: Front Plant Sci. 2023 May 12;14:1176156. doi: 10.3389/fpls.2023.1176156 (PMC10214837; doi:10.3389/fpls.2023.1176156)
Supplement: Supplementary file 1 [file Table_1.docx]

Supplementary Material

Low canopy temperature and high stomatal conductance contribute to high grain yield of contrasting *Japonica* rice under aerobic conditions

Wenliu, Gong^*^, Christopher Proud, Shu Fukai, and Jaquie Mitchell

*** Correspondence:** Corresponding Author: Wenliu Gong, wenliu.gong@uqconnect.edu.au

# Supplementary Figures and Tables

S Table 1 The mean plant height (PH, cm), days to heading (DTH), grain yield (GY, t ha^-1^), grain number m^-2^ (GNM), canopy temperature depression (CTD, ℃) in well-watered experiment (WW20), selection group of 38 genotypes and inclusion in 15 genotype subset indicated.

| Genotype | PH | DTH | GY | GNM | CTD mean | Selection | subset |
| --- | --- | --- | --- | --- | --- | --- | --- |
| 55A |  |  |  |  |  | Checks | √ |
| Apo | 127 | 124 | 8.46 | 45377 | -5.83 | Checks | √ |
| Takanari |  |  |  |  |  | Checks | √ |
|  |  |  |  |  |  |  |  |
| Cocodrie | 77 | 98 | 8.98 | 44854 | -8.42 | High | × |
| Dellrose | 87 | 103 | 10.74 | 53252 | -8.84 | High | × |
| Fado | 82 | 101 | 7.33 | 25675 | -7.74 | High | × |
| Gavina | 64 | 84 | 11.45 | 42125 | -7.89 | High | √ |
| Irat109 | 105 | 99 | 7.16 | 25183 | -8.27 | High | √ |
| Kyeema | 112 | 103 | 7.16 | 37889 | -8.13 | High | × |
| L202 | 87 | 99 | 10.37 | 46996 | -7.87 | High | × |
| Lagrue | 81 | 94 | 9.5 | 45671 | -8.05 | High | × |
| Langi_(Y1) | 92 | 102 | 6.76 | 32358 | -7.73 | High | √ |
| Lemont_(Y2) | 90 | 98 | 11.19 | 47557 | -8.52 | High | √ |
| M205 | 79 | 96 | 9.9 | 40715 | -8.02 | High | √ |
| Paragon | 86 | 110 | 9.51 | 48326 | -8.21 | High | × |
| Rosemont | 116 | 100 | 9.84 | 50348 | -9.1 | High | √ |
| Sherpa | 73 | 83 | 10.05 | 44558 | -7.54 | High | × |
| WAB450-24-3-2-P18-HB | 108 | 99 | 7.15 | 31272 | -7.37 | High | × |
| YRF210 | 100 | 99 | 9.45 | 39842 | -9.78 | High | √ |
| YRL123 | 91 | 102 | 9.24 | 43413 | -8.33 | High | × |
| YRL37 | 94 | 104 | 9.41 | 47312 | -8.37 | High | × |
| YRM59 | 79 | 86 | 8.32 | 36394 | -7.93 | High | × |
|  |  |  |  |  |  |  |  |
| Arlesienne | 97 | 82 | 8.21 | 25463 | -5.56 | Low | √ |
| Baru | 82 | 80 | 4.51 | 21136 | -5.74 | Low | × |
| Bengal | 78 | 99 | 9.16 | 42988 | -5.72 | Low | √ |
| Bogan | 88 | 103 | 7.34 | 32900 | -6.16 | Low | × |
| Calrose76 | 75 | 101 | 10.2 | 39594 | -6.10 | Low | × |
| IR65600-129-1-1 | 89 | 100 | 7.56 | 29279 | -6.11 | Low | × |
| Longdao3 | 92 | 102 | 7.45 | 30602 | -5.92 | Low | × |
| M202_(Y2) | 79 | 104 | 8.89 | 36185 | -6.37 | Low | × |
| Oirase | 110 | 87 | 9.84 | 41684 | -6.10 | Low | × |
| Quest_CT19 | 77 | 95 | 9.12 | 34950 | -6.27 | Low | × |
| Reiziq | 97 | 99 | 10.48 | 44064 | -6.49 | Low | √ |
| Rexoro | 99 | 103 | 7.35 | 37745 | -5.77 | Low | √ |
| Rico1 | 97 | 83 | 6.31 | 23765 | -5.79 | Low | × |
| Tachiminori | 105 | 100 | 5.75 | 25416 | -6.07 | Low | √ |
| Toro2 | 106 | 110 | 6.31 | 42531 | -5.91 | Low | × |
| YRM52 | 84 | 85 | 9.05 | 35788 | -6.13 | Low | × |

S Table 2 The mean, high canopy temperature depression (CTD) group mean (n=19), low CTD group mean (n=16), heritability (H^2^), and correlation between GY and days to heading (DTH), plant height (PH, cm), thousand seed weight (TSW, g), spikelets per panicle (SPP), per panicle weight (PPW, g), panicle number m^-2^ (PNM) and plant density m^-2^ (PD) in well-watered (WW21) and intermittent water deficit (IWD21) experiments ( ns>0.05, *<0.05, **<0.01)

|  |  | DTH | PH | TSW | SPP | PPW | PNM | PD |
| --- | --- | --- | --- | --- | --- | --- | --- | --- |
| WW21 | Mean | 97** | 86.53** | 21.30** | 93.63** | 1.96** | 500.94** | 104** |
| IWD21 |  | 100** | 71.66** | 20.51** | 65.36** | 1.31** | 502.57** | 103* |
|  |  |  |  |  |  |  |  |  |
| WW21 | High CTD mean | 95ns | 84ns | 21.37ns | 100ns | 2.10ns | 497ns | 102ns |
|  | Low CTD mean | 96 | 90 | 21.86 | 89 | 1.89 | 479 | 107 |
|  |  |  |  |  |  |  |  |  |
| IWD21 | High CTD mean | 99ns | 70ns | 20.42ns | 67.77ns | 1.43ns | 512ns | 103ns |
|  | Low CTD mean | 100 | 73 | 20.98 | 61.87 | 1.12 | 489 | 101 |
|  |  |  |  |  |  |  |  |  |
| WW21 | H^2^ | 0.95 | 0.73 | 0.97 | 0.86 | 0.77 | 0.75 | 0.58 |
| IWD21 |  | 0.92 | 0.85 | 0.91 | 0.64 | 0.61 | 0.63 | 0.38 |
|  |  |  |  |  |  |  |  |  |
| WW21 | Correlation between GY and | -0.20ns | -0.29ns | -0.06ns | 0.21ns | 0.22ns | 0.59** | -0.24ns |
| IWD21 |  | -0.24ns | -0.18ns | -0.33* | 0.39* | 0.36* | 0.28ns | -0.18ns |
